# How do lifestyle choices affect the link between living alone and psychological distress in older age? Results from the AgeHeaPsyWel-HeaSeeB study

**DOI:** 10.1186/s12889-020-08870-8

**Published:** 2020-06-05

**Authors:** Razak M. Gyasi, Kabila Abass, Samuel Adu-Gyamfi

**Affiliations:** 1 Aging and Development Unit, African Population and Health Research Center, Manga Close, Off-Kirawa Road, P. O. Box 10787, Nairobi, 00100 Kenya; 2 Department of Geography and Rural Development, Kwame Nkrumah University of Science and Technology, Kumasi, Ghana; 3 Department of History and Political Studies, Kwame Nkrumah University of Science and Technology, Kumasi, Ghana

**Keywords:** Living alone, Psychological distress, Lifestyle choices, Older people, Physical activity, Social participation

## Abstract

**Background:**

Social isolation is widespread and strongly associated with worsening health-related outcomes across the life-course. Despite this broad base of knowledge, there is a paucity of research on the *interactive* effect of lifestyle choices and living arrangements on later life psychological state particularly in low- and middle-income settings. The aim of this study is to examine the influence of living alone on psychological distress in older people and to explore the protective roles of social participation and physical activity participation.

**Methods:**

We used cross-sectional data from the 2016—17 Aging, Health, Psychological Well-being and Health-seeking Behavior Study (AgeHeaPsyWel-HeaSeeB) involving a representative sample of 1200 adults aged 50+ years in Ghana. The study focused on a latent measure of Kessler Psychological Distress Scale (K10) and on the General Practice Physical Activity Questionnaire (GPPAQ). Ordinary Least Squares (OLS) regression models evaluated the interactive effects of living arrangements and lifestyle choices on the K10 score.

**Results:**

Living alone was independent predictor of psychological distress in the overall sample, among females, urban dwellers and all age groups. However, lifestyle choices of physical activity and social participation significantly moderated these associations. Moreover, in the stratified analysis, physical activity moderated the association for males, rural-dwellers and those 65+ years whilst social participation moderated the association for females, urban-dwellers and those 50–64 years.

**Conclusions:**

Lifestyle choices i.e. social participation and physical activity, and demographic factors i.e. age, gender, and residential status strongly attenuate the positive association of living alone with the risk of psychological distress in older age. These findings may inform intervention initiatives targeted at improving mental health of chronically detached and isolated older people.

## Background

Advances in public health, together with improvements in clinical interventions, have led to an increase in life expectancy in almost all regions and territories of the world. This has resulted in major demographic transitions, and it is expected to continue. Between 2015 and 2050, the global population of people aged 60 years or over is projected to almost double, reaching around 2.1 billion [1]. The number of older people residing in sub-Saharan Africa is projected to reach 161 million by 2050 [1] and many of these individuals may live alone and perhaps become socially isolated at some stage because they will outlive their partners or faced with intractable life events such as retirement, daily activity limitations and gradual social change [2, 3].

Living alone has been linked to mental disorders including cognitive function and psychological state particularly in later life [4]. The complexities and crises in later life and the implications for mental health and well-being of older people are strongly related to poor and declining social relationships [5, 6]. In the general population, the presence of quality social relationships has been shown to present numerous physical, physiological and mental health benefits and also increase longevity [6, 7]. For many older people, living alone and the absence of social ties have been associated with worsening self-rated health and all-cause mortality [8].

Indeed, co-residence and the associated social networks may strengthen mental functioning through access to resources, shared decision making, receipt of emotional support, modeling positive health behaviors and coping mechanisms [9]. Accumulating research, however, has shown that living alone and social integration deficit can result in adverse immune responses and mental distress in later life [10]. Typically, various studies have shown how social networks and intergenerational support particularly for older people are strongly embedded in the Ghanaian sociocultural structure [11]. Living alone, especially, as a result of social ostracism and widowhood is, thus, seen as a critical public health and socio-emotional problem and presents far reaching implications for both mental and physical health outcomes during older age.

Social ties may be strengthened or newly formed in older age which may modulate social isolation contexts including living alone. Co-residence or having a strong, supportive networks differentiated by age, gender and residential status may provide important benefits for mental health through shared or powered decision making, survivorship care planning, psychosocial well-being and later life functional independence [2, 9]. Insights from the analysis of the linkages between living alone and later life mental disorder in a sub-Saharan African setting where the intersection of aging and social change is occurring rapidly can create a more robust understanding of how social circumstances may influence well-being, survival and later life social policy discourse. Very importantly, social participation and regular moderate-to-vigorous physical activity often decline with age chiefly due to the declines in the activities of daily living (ADL) and socioeconomic disengagements [12]. Although these mechanisms have been reported to predict mental health outcomes [6, 13], their moderating effects in the relationship between living alone and later life mental disorder risks remain much less explored.

There has been an incessant call for holistic action to identify potential mechanisms that explain the association of living arrangements with mental health outcome in older adults [4, 14–16]. Investigating how lifestyle choices such as physical activity and social participation modify the association of living alone with mental disorder is potentially relevant for critical health policy and public health interventions. The paper examines how living alone impacts psychological distress among community-dwelling older Ghanaians and to explore the moderating effects of physical activity and social participation in this link. We hypothesize that (1) the odds of psychological distress will be higher for older people who live alone; (2) lifestyle choices will attenuate the severity of psychological distress among isolated older people.

## Methods

### Data and sampling

Data for this analysis were drawn from a 2016–17 Aging, Health, Psychological Well-being and Health-seeking Behavior (AgeHeaPsyWel-HeaSeeB) Study which was conducted in Ghana. This study applied a probability-based sample consisting of community-residing adults, aged ≥50 years. We employed a multi-stage stratified cluster sampling procedure in this study and details of the selection technique have been reported in previous works [2, 6, 14, 17]. The key eligibility requirements were that the participants were at least 50 years of age, resident in the respective study areas and had lived in the study setting for the past 2 years. This was important to exclude potential participants who were in transition (such as short-lived visitors and migrants) who may not have a clear-cut living arrangements at the time of the study*.*

The sample size was estimated using a formula, $$ n= design\ effect\times \left[{\left({Z}_{\raisebox{1ex}{$\alpha $}\!\left/ \!\raisebox{-1ex}{$2$}\right.}\right)}^2\times P\left(1-P\right)\right]/{\varepsilon}^2 $$, [18] assuming 5% margin of error, 95% confidence interval, 1.5 design effect, 5 and 15% of type 1 and type 2 errors respectively, and a conservative estimation or default prevalence of 50% (because the actual proportion of people aged 50+ years in the selected areas was unknown). In the selection process, 1247 older persons were selected by systematic random sampling with the sampling interval varying by relative size of the study communities. Of the 1247 approached, 1219 (97.8%) were eligible to participate. Of these eligible participants, 19 declined to participate in the study yielding an overall participation rate of 98.4% (*N* = 1200). The statistical power calculation revealed that the sample size had 85% power to detect an odds ratio of ≥2. The survey questionnaire was developed in English, translated into Asante Twi (the principal dialect in the study area) and back translated into English with reconciliation of discrepancies for quality control of the translation procedure following WHO translation guidelines for assessing instruments [19]. Face-to-face interviews were conducted using interviewer-administered questionnaires, taking into consideration the high illiteracy rate among the sample.

### Measures

#### Dependent variable

We characterized psychological distress using the Kessler Psychological Distress Scale (K10) [20]. This scale was developed as a screening tool for psychological distress in the general population. The K10 distinguishes Diagnostic and Statistical Manual of Mental Disorders, 4th Edition (DSM-IV) from non-cases [20] and is strongly associated with the Composite International Diagnostic Interview (CIDI) diagnosis of anxiety and affective disorders. The K10 scale consists of 10 items measuring level of anxiety and depression in the previous 4 weeks. Each item has five responses 1 = none of the time, 2 = little of the time, 3 = some of the time, 4 = most of the time and 5 = all of the time. A total score ranges from 10 to 50, and higher scores reflect higher psychological distress.

#### Independent variable

Living arrangements were assessed with an item: “Do you live with anyone else?” with four mutually exclusive categories 1 = live alone (live alone in a one-person household), 2 = with spouse only (live with a spouse in a two-person household), 3 = with children (live with any of their children i.e. sons, daughters, children-in-law and step/adopted/foster children) and 4 = with others. Because older people who live alone are characteristically distinct across social, economic and health conditions compared to those in co-residence (who are likely to receive some support) [4], we dichotomized the responses with 0 = *co-residence* and 1 = *living alone*.

#### Moderating variables: lifestyle choices

Two important lifestyle behavior variables were considered as the potential moderating variables based on previous literature. First, physical activity has been linked to lower levels of social exclusion among older people [12, 13]. This was assessed with the General Practice Physical Activity Questionnaire (GPPAQ) [21]: “How many days in the last week did you walk for at least 30 minutes in total?”; “do moderate activities such as dancing for about 30 min in total?”; “do vigorous activities such as running, sporting, gardening, and heavy housework?” The responses were recorded on a continuous scale (ranging 0–21) with higher scores indicating physically active. Second, social participation in neighborhood activities was assessed with the item: “How often in the last one month have you attended social activities including family meetings, religious services, social clubs or organization meetings, sports or cultural activities and civic or political organizations meetings?” on a five-point response scale (0 = never, 1 = less frequently, 2 = frequently, 3 = very frequently, 4 = every day). For the purposes of dichotomization, we transformed the responses into 0 = not frequently (never/less frequently,) and 1 = frequently (frequently/very frequently/every day).

#### Confounding variables

Potential confounders were identified and selected based on theoretical assumptions and empirical findings of past literature [3, 22] suggesting their respective impacts on psychological distress and mental health in general. The socioeconomic covariates included sex (male or female), age (50–64 years or ≥ 65 years), spatial residence (rural or urban), employment status (unemployed or employed) and level of education (primary school/no attendance, secondary education or higher) and individual monthly income. Loneliness was assessed based on the Three-Item Loneliness Scale of the University of the California, Los Angeles (UCLA) : How often do you feel you lack companionship? How often do you feel left out? How often do you feel isolated? (hardly ever/never, some of the time/sometimes or often/always) with an overall score 3–9. Higher scores indicate higher levels of loneliness [23]. In the present study, Cronbach’s alphas for loneliness was α = 0.78.

In terms of health-related factors, self-rated health was assessed with a question “In general, how would you rate your health?” using four-level responses (very good, good, fair or poor) whilst chronic illnesses included the diagnoses by a health care professional of 10 illnesses (hypertension, diabetes, respiratory diseases, cancers, stroke, chronic kidney diseases, asthma, arthritis, depression and insomnia). Functional status was assessed with five-item of basic activities of daily living (ADL) that are required to take care of oneself and also commonly used to gauge older people’s daily performance such as bathing, using the toilet, eating, dressing and getting in and out of bed (not limited at all, less limited, somewhat limited or much limited). A sum-score was estimated (range 5–20), with higher scores reflecting poorer functional status [24].

### Statistical analysis

Sample characteristics and bivariate estimations were calculated to describe the study sample. Multiple linear regressions were used to analyze the association between living alone and mental disorder. In addition, studies contend that social isolation and mental health factors may vary by gender, age and spatial differences [2]. We, therefore, performed regressions stratified by gender, age and rural/urban residential status to further investigate the role of these variables in the association. In additional analyses, the moderating roles of physical activity and social participation in the association between living alone and mental health were tested in terms of the overall sample and gender, age and residential specific examinations. The statistical significance was determined with *p* < 0.05. Data analyses were performed using IBM-SPSS Statistics for Windows application (version 21; Chicago, IL, USA). In all regressions, multicollinearity was tested using the variance inflation factor (VIF). The largest variance found was 2.96, suggesting that a problem with multicollinearity was not present [25]. We reported odds ratios (ORs) and their corresponding lower and upper 95% confidence intervals (CIs) by computing an exponentiation of the regression coefficients to obtain each corresponding OR. This is because, the exponential function of the regression coefficient is the odds ratio associated with a one-unit increase in the exposure [26, 27].

## Results

### Descriptive statistics

A total of 1200 people 50 years or older completed the AgeHeaPsyWel-HeaSeeB eligibility survey. Of this, 458 (38.2%; 95%CI: 35.4–41.0%) lived alone and the mean score of mental disorder was 13.54 [SD = 5.10] (Table 1). The overall mean age of participants was 66.15 years [SD = 11.85 years] with a range from 50 to 111 years. Participants were predominantly females (63.3%), lived in urban areas (55.0%), had lower educational levels (86.2%) and were not employed (55.6%) which reflected in lower and highly diverse income levels (¢308.180 [SD = 338.893]). Moreover, 55.2% felt lonely, 95.3 and 73.3% respectively maintained regular contact with family and participated in social events whilst one-half of the respondents engaged in physical activity. The mean functional impairment was 13.70 [SD = 5.09], nearly one-half self-reported worsening health, and 53.0% were diagnosed with at least one chronic illness. In the bivariate analysis, living alone was significantly associated with decreasing age (50–64 age group), male gender, urban dwelling, lower education, the employed, income levels, loneliness, lower income level, physical inactivity, self-rated poor health, less frequent social participation, poor functional status, living with chronic condition and psychological distress status (*p* < 0.001) (Table 1).

**Table 1:** Descriptive and bivariate analysis of study variables

Variable	Living arrangements						*P*-value
	Overall		Co-residence		Living alone		
	N	(%)	N	(%)	N	(%)	
Total	1200	(100)	742	(61.8)	458	(38.2)	–
Age (years)							
50–64	585	(48.8)	304	(41.0)	281	(61.4)	< 0.001
65+	615	(51.3)	438	(59.0)	177	(38.6)	
Gender							
Female	759	(63.3)	563	(75.9)	196	(42.8)	< 0.001
Male	441	(36.8)	179	(24.1)	262	(57.2)	
Residence							
Rural	540	(45.0)	366	(49.3)	174	(38.0)	< 0.001
Urban	660	(55.0)	376	(50.7)	284	(62.0)	
Educational level							
Primary or none	1034	(86.2)	672	(90.6)	362	(79.0)	< 0.001
Secondary	104	(8.7)	48	(6.5)	56	(12.2)	
Tertiary	62	(5.2)	22	(3.0)	40	(8.7)	
Employment status							
Unemployed	667	(55.6)	486	(65.5)	181	(39.5)	< 0.001
Employed	533	(44.4)	256	(34.5)	277	(60.5)	
Monthly income (¢) [M(SD)]	308.180	[338.893]	410.3	[469.6]	239.7	[180.4]	< 0.001
Loneliness							
Not lonely	538	(44.8)	253	(34.1)	285	(62.2)	< 0.001
Lonely	662	(55.2)	489	(65.9)	173	(37.8)	
Physical activity							
Not-active	594	(49.5)	426	(57.4)	168	(36.7)	< 0.001
Active	606	(50.5)	316	(42.6)	290	(63.3)	
Frequent family contacts	1143	(95.3)	699	(94.2)	444	(96.9)	0.030
Frequent social activity	880	(73.3)	535	(72.1)	345	(75.3)	0.220
Self-assed health							
Very good	239	(19.9)	109	(14.7)	130	(28.4)	< 0.001
Good	369	(30.8)	216	(29.1)	153	(33.4)	
Fair	348	(29.0)	239	(32.2)	109	(23.8)	
Poor	244	(20.3)	178	(24.0)	66	(14.4)	
Functional status [M(SD)]	13.70	[5.09]	15.17	[4.86]	12.79	[5.02]	< 0.001
Diagnosis of NCDs	636	(53.0)	416	(56.1)	220	(48.0)	0.007
Psychological distress [M(SD)]	13.54	[5.10]	12.97	[5.04]	14.11	[4.96]	< 0.001

*P*-values are based on *χ*^2^ test and compare the difference by living arrangements (co-residence vs living alone) and independent variables included in the regression models

### Main regression models

The results of the multiple regressions analysis are presented in Table 2. Unadjusted results showed a significant positive association between living alone and psychological distress in the overall sample (OR = 2.435; 95% CI: 1.908–3.106) and in all stratified sub-groups for gender, age and spatial differences. After adjusting for various potential confounders, linear regressions showed that older persons living alone were 1.5 (OR = 1.463; 95% CI: 1.065–2.009) times more likely to experience mental distress in the total sample. This model accounted for 27% of the explained variance in the mental disorder outcome. Stratified analysis showed that females (OR = 1.630; 95% CI: 1.074–2.474), urban dwellers (OR = 1.699; 95% CI: 1.129–2.557), those aged 50–64 years (OR = 2.064; 95% CI: 1.348–3.160) and 50–64 years (OR = 1.403; 95% CI: 1.051–2.478) who lived alone had higher odds of experiencing mental distress but not among males and rural inhabitants (Table 2).

**Table 2:** Associations between living alone and psychological distress: OLS Regression Models

Variables	MODEL 1		MODEL 2	
	OR	(95% CI)	OR	(95% CI)
Potential confounders	√		√	
Main model: Living arrangements: Living alone vs co-residence	2.435	(1.908–3.106)***	1.463	(1.065–2.009)**
Stratified models				
Gender				
Female	2.448	(1.743–3.439)***	1.630	(1.074–2.474)*
Male	2.021	(1.364–2.994)***	1.122	(0.657–1.916)
Age				
50–64	3.184	(2.265–4.475)***	2.064	(1.348–3.160)***
65+	1.964	(1.364–2.827)***	1.403	(1.051–2.478)**
Residence				
Rural	2.511	(1.719–3.667)***	1.415	(0.811–2.466)
Urban	2.359	(1.711–3.252)***	1.699	(1.129–2.557)*
*N*	1200		1200	

OR is the odds ratio; CI in parenthesis is confidence interval; √ represents potential confounders

Model 1: Unadjusted model

Model 2: Adjusted model included living arrangements, age, gender, residence, education level, employment, income level, family contacts, social participation, loneliness, physical activity, self-reported health, functional status, and diagnosis of chronic diseases

Stratified models included Gender (Male vs Female); Age (50–64 vs 65+) and Residence (Rural vs Urban)

*p < 0.05; **p < 0.005; ***p < 0.001

### Moderated regression models

**Fig. 1:**
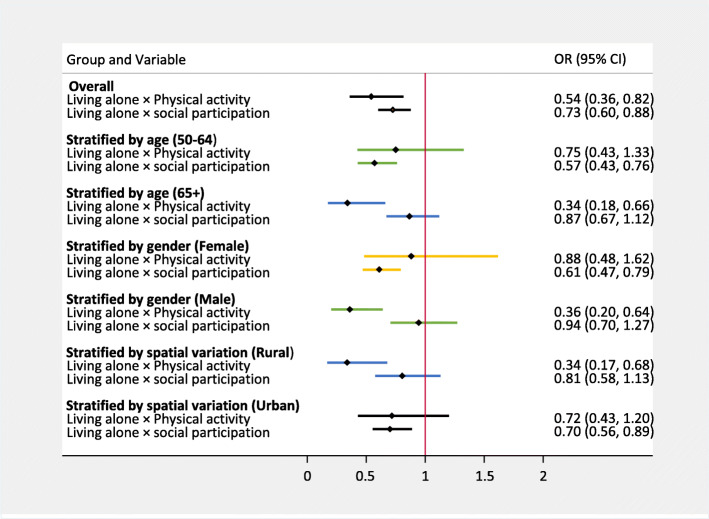
Overall sample, age-, gender- and spatial location-specific associations and moderating factors in the association of living alone and psychological distress. OR is the odds ratio; CI in parenthesis is confidence interval. All Models were adjusted for age, gender, rural/urban residence, education level, employment, income level, family contacts, loneliness, self-reported health, functional status, and diagnosis of chronic diseases

In addition, it was tested whether physical activity and social participation moderate the association between living alone and psychological distress (Fig. 1). In the total sample, the interaction terms (living alone × physical activity) and (living alone × social participation) significantly attenuated the risk of psychological distress by 46% (OR = 0.543; 95% CI: 0.361–0.816) and 27% (OR = 0.726; 95% CI: 0.601–0.877) respectively among those living alone. Further sensitivity interaction analysis showed similar results among the stratified sub-groups. For example, social participation reduced psychological distress risk by 39% among females and 30% among urban dwellers. Also, physical activity reduced incidence of psychological distress by 64% among males and 66% among rural dwellers who lived alone.

## Discussion

### Main findings

This study of older Ghanaian adults is the first to utilize representative data to investigate and further advance extant literature by testing whether two important lifestyle choices of physical activity and social participation as well as demographic variables independently explain the association between living alone and psychological distress in this population. Results of the multivariable OLS regression analysis revealed that living alone significantly increased the odds of psychological distress among older people. Moreover, this association was moderated by age, gender and residential status. In addition, our findings add to the social relationships and mental health literature by providing evidence of the moderating effect of lifestyle variables of physical activity and social participation on the relationship between social isolation and psychological distress.

### Possible explanations

The findings provide some evidence to support the first study hypothesis suggesting that older people living alone report poorer mental health. Although studies linking living arrangements and psychological distress *per se* is much limited, there is an established literature showing that living alone in later life is strongly linked to poor mental health [17, 28, 29]. Our results are consistent with a number of previous studies reporting significantly higher risks of wide ranging mental disorders such as depressive symptoms, anxiety and declining cognitive function (which generally characterize psychological distress) among older people living alone compared to those living with others [22, 28, 29]. For example, in a population-based sample of 12,647, McKinnon and colleagues [29] found that living alone predicted a 2.3% point higher prevalence of depression among older people in 15 sub-Saharan African countries (including Ghana) in relation to those living with at least one other person.

A number of hypotheses could explain the positive association between living alone and mental disorder. First, living alone is recognized as one of the most stressful later life events which may result from widowhood and social ostracism [2]. These circumstances may potentially lead to negative changes in individuals’ social environment, risk factors for social isolation and loneliness, which may derail mental health outcome. Second, accumulating research demonstrate that the prescription of antidepressant, anxiolytic and hypnotic drugs is higher in people living alone than in those with others [28] which may trigger common mental disorders. Further, whilst perceived social isolation escalates immune dysregulation risks, both immune suppression and activation are key antecedents for depression and other mental disorders [30] particularly in older age. However, our observation is inconsistent with findings emerging from previous studies in the advanced settings in particular indicating that living alone is unrelated to mental disorders [31–33]. The disparity may result from the view that unlike Western societies, African sociocultural landscape reflects strong communally integrated societies and that living alone become a critical condition for older Africans [11]. The myriad of measurement scales for aspects of mental health might have also contributed to diverse findings. Future research should investigate the specific pathways through which living alone influences mental health in general and psychological distress in particular.

In addition, our stratified analysis demonstrated that living alone was independently associated with higher risk of psychological distress in older women and urban dwellers but this association was not established for men and rural counterparts. These differences may relate well to the different gender roles, and sociocultural coloration between rural and urban African settings. Generally, whilst women are more amiable to social relationships compared to men, circumstances of living alone may likely cause more stress to women and influence their psychological state. In the African traditional context, rural dwellers are generally bonded and closely related to one another [11, 14]. Individuals living alone in such circumstances may easily get attached with other community members unlike the urban areas where people mostly “mind their businesses” and hardly connect and share thoughts with others leading to a higher chance of experiencing mental disorders.

More importantly, our hypothesis regarding the possible modifying effects of buffering resources in the association of living alone with psychological distress was supported. The moderation effects of subjectively assessed physical activity and social participation as core elements of neighborhood dimensions were strongly demonstrated. There are several possible mechanisms through which neighborhood social quality might modify psychological distress and living alone interrelationship. Social ties can buffer stressful and adverse life events and, therefore, counter the onset of mental illness, and also moderating their negative impacts. Social isolation and a lack of social participation can underscore various mental health challenges among older cohorts [33].

Good neighborhood social quality may increase the availability of social activities. Demonstrated by the *convoymodel* *of social relations*, participation in social events may allow older people who live alone the opportunity to meet new people and form social networks which may decrease feelings of loneliness and mental disorder [34]. Social networking and the concomitant practical help from relevant others make older people feel safer and more secure. This alleviates the stress associated with living alone and its consequence of mental disorders [17, 35]. The ability to maintain a sense of belonging with family, close friends and participation in social or community events appears to buffer the negative affect and emotional suffering when living alone. Similarly, intensifying physical activity and engagement in group leisure-time activities can modulate mental problem among older adults. Indeed, the stress buffering hypothesis of physical activity suggests a mechanism to reduce stress and also helps to improve mental health. Physical activity and social participation perhaps establish a stress-focused behavioral coping for wide-ranging mental problems including psychological distress, mood, depression, loneliness, and anxiety [2, 13]. These findings reinforce previous research demonstrating the role of social participation and physical activity in tempering mental problems [2, 12].

### Strengths and limitations

Being one of the first to evaluate the *interactive* impacts of lifestyle choices and living arrangements on psychological distress among older adults in an innovative context, the present analysis draws strength from relatively large and nationally representative sample achieved by pooling proportionately selected participants from across rural and urban settings. Moreover, psychological distress outcome and neighborhood physical activity was quantified using validated scales with very good psychometric properties. However, the retrospective self-reported measures and cross-sectional design used meant that, whilst recall biases become highly inevitable, directionality and causal conclusions cannot be made. Although this limitation is recognized in other studies using similar design, evidence for the validity of self-reported data and non-longitudinal designs has been demonstrated in previous studies. Future research on the linkages between living arrangements and mental health in sub-Saharan Africa should usefully explore longitudinal data that may reveal temporal relationships among variables.

## Conclusions

Among older people in a sub-Saharan African country context, the findings of this study underline the premise that living alone increases the risks of psychological distress with marked demographic disparities. Typically, this association strongly reflects in those aged 50–64 years, among women and urban dwellers. Importantly, lifestyle choices of social participation and physical activity moderate the relationship. Our study emphasizes the need to consider social and physical activities for isolated older adults as innovative interventions to improve mental health and healthy aging. Critical gerontological research and environmentally driven initiatives including older age-friendly neighborhood, community development and social programs may promote interpersonal relations toward improved psychological functioning for older people.

## Data Availability

The datasets used and/or analyzed during the current study are available from the corresponding author on reasonable request.

## Abbreviations

ADL: Activities of Daily Living
CIDI: Composite International Diagnostic Interview
DSM-IV: Diagnostic and Statistical Manual of Mental Disorders, 4th Edition
GPPAQ: General Practice Physical Activity Questionnaire
WHO: World Health Organization
UNDESA: United Nations Department of Economic and Social Affairs
